# Optimizing Precision Medicine for Public Health

**DOI:** 10.3389/fpubh.2019.00042

**Published:** 2019-03-07

**Authors:** Gemma A. Bilkey, Belinda L. Burns, Emily P. Coles, Trinity Mahede, Gareth Baynam, Kristen J. Nowak

**Affiliations:** ^1^Office of Population Health Genomics, Public and Aboriginal Health Division, Department of Health, Government of Western Australia, Perth, WA, Australia; ^2^Office of the Chief Health Officer, Public and Aboriginal Health Division, Department of Health, Government of Western Australia, Perth, WA, Australia; ^3^Genetic Services of Western Australia, King Edward Memorial Hospital, Department of Health, Government of Western Australia, Perth, WA, Australia; ^4^Western Australian Register of Developmental Anomalies, King Edward Memorial Hospital, Department of Health, Government of Western Australia, Perth, WA, Australia; ^5^Faculty of Health and Medical Sciences, School of Biomedical Sciences, The University of Western Australia, Perth, WA, Australia; ^6^Harry Perkins Institute of Medical Research, Queen Elizabeth II Medical Centre, Perth, WA, Australia

**Keywords:** genomics, public health, policy, precision medicine, genomic testing, molecular diagnostics, genetic disease, genetic therapies

## Abstract

Advances in precision medicine have presented challenges to traditional public health decision-making paradigms. Historical methods of allocating healthcare funds based on safety, efficacy, and efficiency, are challenged in a healthcare delivery model that focuses on individualized variations in pathology that form the core of precision medicine. Public health policy and decision-making must adapt to this new frontier of healthcare delivery to ensure that the broad public health goals of reducing healthcare disparities and improving the health of populations are achieved, through effective and equitable allocation of healthcare funds. This paper discusses contemporary applications of precision medicine, and the potential impacts of these on public health policy and decision-making, with particular focus on patients living with rare diseases and rare cancers. The authors then reconcile these, presenting precision public health as the bridge between these seemingly competing fields.

## Introduction

Precision medicine has catalyzed strong debate over the merits and realities of a more personalized approach to healthcare. In one camp are the ideological, who believe that the utopia of medicine can exist in a world where patients' genomes, and -omics related information such as exposomics and metabolomics, can guide real-time, individualized prevention and therapeutics for improved outcomes to all (1, 2). In the other camp are those who tell a cautionary tale, citing current inability to reconcile the dream with the reality (3, 4). In particular, there is concern about the relevance and impact of individualized precision medicine approaches for public health, where populations are the traditional focus for intervention and decisions for which healthcare initiatives to fund must be rationalized within finite budgets.

Precision public health (PPH) is an emerging focus of public health that complements the development of precision medicine and utilizes advances in new technologies and knowledge unlocked through big data to better target public health efforts within populations (5). There has been increasing global interest in this approach, with the White House and Bill and Melinda Gates Foundation sponsoring a 2016 conference entitled “Precision Public Health: The First 1,000 Days.” Additionally, the Western Australian Department of Health and the Rockefeller Foundation hosted two separate international events on PPH in 2018 (6).

One of the many potential roles of PPH is to use population level data to better identify how individuals can be aggregated into larger groups. This could be achieved using the increased knowledge derived from precision medicine about the biological pathways involved in disease. Such an approach may be critical to ensuring that evidence-based research methodologies can still inform decision-making in the context of increasingly smaller target groups for therapies and diagnostics. In addition, a PPH approach, which is grounded in the public health values of whole population health improvement and equity, is seen as a safeguard against the potential “blind optimism” which can surround new technology (5). Herein, we provide an overview of key precision medicine initiatives, and consider how applying a PPH approach can ensure that precision medicine can be safely, effectively and equitably delivered for the benefit of the population. We illustrate this concept using examples from the fields of rare diseases and uncommon cancers, noting that the same approach could be applied in other areas of medicine.

Challenges will be faced in bringing precision medicine safely to the population. In particular, genomic technological advances and subsequent utilization of precision medicine within healthcare has given rise to unique ethical considerations (7–9). Such considerations present challenges to healthcare providers, governments and policymakers to provide assurance for patients and the population that privacy, testing, return of results and data storage are conducted in an ethical way. There are many papers that explore these considerations (7, 8, 10–12), and as such, these ethical issues will not be discussed in detail within this paper.

## The Contribution of Genetic Tests and Therapies to Precision Medicine

Advances in the development of genetic tests and therapies provide the potential to transform medicine and create unprecedented ability for detection, prevention, and treatment of diseases. Therapy approaches based on genetic variants and specific biomarkers have been increasing over the last few decades in association with the increasing availability and affordability of genomic sequencing technology. In this context, there has been growing interest in and advocacy for precision medicine approaches (13). This interest is highlighted by the World Economic Health Forum's Precision Medicine Programme, which “aims to support the development of policy frameworks and governance protocols to realize the societal benefits, and mitigate the risks from, precision medicine” (14).

Consideration of individual level variation, of both the person and/or their disease, is at the heart of precision therapies. For example, tumors have been eloquently described as “malignant snowflakes,” which articulates that no two cancers will have the same molecular profile (15). Subsequently, therapeutic regimens must consider this inevitable variation in disease, with an individualized therapeutic approach likely to produce better health gains (15). Similarly, there are thought to be up to 6,000 rare diseases, many of which have underlying genetic causes and which may require different therapeutic approaches. Furthermore, genetic variants have been shown to influence metabolism of drugs and a range of drugs include information on their labels about adverse drug reactions or different dose recommendations based on a person's genomic profile (16, 17). It is possible that an individual's genomic information could be used to rationalize and guide therapeutic options and dosing at the point of prescribing. However, the approval and use of such precision therapies is often reliant on “companion” diagnostic tests that are able to identify who is likely to benefit from a particular medicine, requiring parallel mechanisms of assessment and regulation for diagnostic and therapeutic approaches. Some recent examples of precision therapies and interventions are explored below.

### Biomarker Specific Therapies

In 2017, the Food and Drug Administration (FDA) of the United States of America (USA) approved more precision medicines and companion tests compared to any prior year (18). One example was the approval of pembrolizumab (Keytruda®), which marked the first solid cancer therapy approved for use based on the presence of a specific biomarker rather than a tumor's location (19). Similarly, trastuzumab-dskt (Ogivri™) was approved as the first biosimilar agent, targeting both stomach, and breast tumors overexpressing the *HER2* gene, possibly facilitating competition and aiding lower healthcare costs (20).

As these tests are dependent on the presence of specific biomarkers, they are therefore reliant on companion genetic tests. Two examples of companion tests are MSK-IMPACT™ (screens 468 genes) and FoundationOne CdX™ (screens 324 genes), both solid tumor tests and the first massively parallel sequencing *in vitro* diagnostic tests. Both tests screen multiple oncogenes to identify variants that might assist in the clinical management of patients, and identify patients with certain tumor types who may benefit from approved targeted treatment options (21, 22).

### Genetic Therapies

Significantly, three of the 2017 FDA approvals were the first gene therapies ever approved by the FDA, including voretigene neparvovec (Luxturna™) for retinal dystrophy, the first to treat an inherited disease. Spark Therapeutics gave Luxturna™ a list price of US$425K per eye, making it the most expensive medicine in the USA per dose (23).

The FDA also gave fast track designation and priority review in 2016 for two orphan drugs for genetic neuromuscular diseases (both antisense oligonucleotides), representing significant advances in the treatment of rare diseases. In September 2016, the FDA provided accelerated approval for eteplirsen (Exondys 51™) for Duchenne muscular dystrophy (24), and nusinersen (Spinraza®) was approved in late December for early fatal spinal muscular atrophy (25). Both these treatments need to be delivered for the remainder of a patient's life. Exondys 51™ costs around US$300K per patient per year, and in the second quarter of 2018 it generated Sarepta Therapeutics over US$73 million in net revenue (26). Spinraza® has a list price of US$125K per injection, translating to US$750,000 in the first year of treatment per patient, and US$375K for each subsequent year. In Australia, Spinraza® was listed on the Pharmaceuticals Benefits Scheme from 1 June 2018 (27), meaning patients pay less than AU$40 per script. However, in August 2018, Britain's healthcare cost agency (National Institute for Health and Care Excellence; NICE) deemed Spinraza® too expensive, and its long-term effectiveness too uncertain, for routine use within the National Health Service [NHS; (28)].

### Genetic Editing

Presently, a strong focus for precision therapies is on genome editing or engineering, with greatest emphasis on three genome-modifying techniques all harnessing programmable nucleases, which can be considered “molecular tools.” These are CRISPR-Cas9 (clustered, regularly interspaced, short palindromic repeats—CRISPR; CRISPR-associated protein 9—Cas9); zinc finger nucleases (ZFNs); and transcription activator-like effector nucleases (TALENs). All of these nucleases have been translated to patient care to some degree.

TALEN engineered cells were first applied to patients with B-cell acute lymphoblastic leukemia (B-ALL) (29). Extremely promising trial outcomes led to the drug tisagenlecleucel (Kymriah®) gaining FDA-approval in August 2017, with further approval in May 2018 for use with large B-cell lymphoma (30–32). In the European Union, tisagenlecleucel was approved for B-ALL in August 2018, and less than a fortnight after, the NHS England made a commercial arrangement with the drug's maker Novartis to provide the drug to children with advanced leukemia (33).

In November 2017 as part of a phase 1/2 trial, the first human had ZFN gene editing tools injected into their bloodstream, in an attempt to treat the patient's previously incurable, rare metabolic disease [Hunter syndrome; (34)]. Other trials harnessing ZFN technology are also underway [e.g., severe hemophilia B (35), mucopolysaccharidosis I (36) and transfusion-dependent beta-thalassemia (37)]. Multiple enticing reports have emerged of success from CRISPR-Cas9 application for disease treatment, prevention or reversal in preclinical models, e.g., with mouse [e.g., embryo (38) and postnatal (39) delivery], and dog (40) models of Duchenne muscular dystrophy. However, the first description of CRISPR-Cas9 gene technology used to correct human embryos (41) with genetic mutations causative of hypertrophic cardiomyopathy has been controversial (42). Yet current clinical trials harnessing CRISPR-Cas9 gene editing technology in adults include those for advanced esophageal cancer (43); leukemia and lymphoma (44); transfusion-dependent beta-thalassemia (45); and relapsed refractory multiple myeloma, synovial sarcoma, and myxoid/round cell liposarcoma (46).

New therapies such as these offer efficacious treatment options to patients with serious, rare conditions, when previously there were none. However, based on current prices, it is unlikely that these diagnostics and therapeutics present viable options to patients or their families, especially on an ongoing basis. Therefore, patients are reliant on governments and health insurers to cover the majority of the cost. Policymakers need to carefully evaluate the test or treatment's affordability, whilst appreciating the additional advantages it might bring to an affected person and the wider population. Additionally, balance is needed when deciding on the pricing of therapeutics to ensure access to excellent health care for patients, whilst also supporting biopharmaceutical innovation and investment into new therapeutics.

## Decision-Making Approaches for MAXIMIZING Population Health

In publically funded healthcare systems two broad priorities for decision-makers are “to do the most, for the most” (47), and to “reduce health inequity” across the population (48). Within the constraints of finite resources, the maximum number of people should receive the maximum benefit from the health programs and therapeutics that are publically funded. In other words, this is the “*n of many”* approach for optimizing population health outcomes. However, decision-making should not exacerbate existing health disparities and targeted investment is often required to address health inequities that emerge through societal mechanisms, including healthcare decision-making mechanisms. To assist with this, decision-makers rely on tools to allow for transparent, fair and reproducible decisions to determine which programs and therapeutics should receive public investment.

The economic evaluation of healthcare initiatives allows decision-makers to evaluate the cost of providing an intervention or therapy, and determine what the outcomes will be if that particular therapy or program is chosen over another (49). In short, it allows decision-makers to seek which outcomes can be “purchased” for the population and at what financial cost. Crucial to this paradigm is the need to evaluate which benefits to the population are foregone when one intervention is chosen at the expense of another (the “opportunity cost”) (50). Ultimately, cost thresholds that determine which programs or therapeutics will be funded are somewhat flexible. However, in situations where interventions are costly or where there is a lack of available evidence for utility or cost-effectiveness, there is a greater reliance on other tools for decision-making, such as the determination of social values and the influence of the political agenda.

The incorporation of social values into decision-making is less defined than economic evaluations, given the inability to attribute a standardized weighted value to social concepts. However, research has occurred to quantify social preferences, although these methods are as yet not widely adopted (49). Examples of such social concepts include whether an intervention targets a population of unmet need; whether the intervention satisfies the “rule of rescue,” i.e., patients for whom there is no other therapeutic option, or whether the program may target a population considered at higher risk such as lower socioeconomic groups. It is for such patients that standardized health care decision-making paradigms become challenged.

An additional consideration for decision-making in the event of unfavorable economic evaluations is the inability to attribute value to political goals for health care, which may catalyse innovation incentives and funding for conditions on the political agenda (51). Uncertainty of leadership, changing agendas and political factions can lead to the reliance on political will, which is arguably the most volatile tool for decision-making. While the window of political will is open, health systems could proceed with haste to sustainably integrate new methods into the delivery of healthcare to better the health of the population.

Therapeutic efficacy and outcomes used in economic evaluation and decision-making are traditionally determined through the results of large randomized controlled trials (RCTs) or systematic reviews of RCTs. Given the reliance of decision-making on large numbers of participants in RCTs, this presents a disadvantage for conditions and populations in which large numbers are difficult to achieve, such as has occurred for patients with rare diseases and uncommon cancers (52). In these situations it is difficult to generate enough data in support for the public funding of therapeutic agents, often leaving this subset of patients without the same therapeutic options as patients with more common conditions (53). This scenario fails the goal of equity of access to care, as a disparity will exist when only those who can privately afford these treatments are able to access them. In the oncology patient population, particularly for those with rare and uncommon cancers, or common advanced cancers where therapeutic options have been exhausted, the cost of an intervention may be high, the outcomes may be relatively poor, and the evidence base may be minimal (54, 55).

Although major challenges still exist with translating precision therapies and companion tests from bench to bedside, such as minimization of off-target effects, cytotoxicity, and immunogenicity, a new frontier in medicine is emerging. In theory it is possible that precision medicine approaches such as gene therapies and gene editing will eventually be capable of targeting most, if not every monogenetic disease. Whilst a suite of underlying technological platforms and their delivery routes could be a common base across most therapies, they could be made bespoke by using specific modifications dependent on the genetic variations, such as different guide RNAs in the case of CRISPR-Cas9 approaches. In such a scenario, not only may precision medicines be designed for small subsets of the population (such as those with a very rare disease), they may indeed be so precise that they are tailored to genetic variations unique to a single family or indeed only to one individual, e.g., an *n of 1*. In this context, traditional decision-making paradigms are challenged, because many population health decision–making approaches, as well as medical research funding models, rely on demonstrating the relevance of interventions to the broader population.

With potentially infinite combinations of therapies and interventions likely to arise from this precision approach, how can we continue to approach healthcare decision-making in a standardized way? In particular, how can this approach provide assurance that therapies are safe (e.g., safety data) when we are facing an infinite number of therapeutic combinations? Additionally, gene editing approaches are likely to be ineffective if applied to individuals without the targeted genetic variant/s, or indeed may create disease through their action. Consequently, efficacy within cells derived from affected patients, or preclinical models created with the same genetic variants, will be necessary yet labor- and cost-intensive. If we require data on safety, efficacy, and economic efficiency for every permutation of therapeutic agents available to precision medicine, how can we best integrate this emerging knowledge at a population level? A similar scenario to that seen for patients with rare diseases is likely to play out for precision medicines unless concerted effort is directed toward equitable and efficient processes.

Many of the lessons learned and approaches used to combat healthcare equity issues in the field of rare diseases and population health will become increasingly important in the move to precision medicine approaches. These lessons include aggregation of individuals or small population cohorts into larger cohorts with specific shared needs, international collaboration and sharing of expertise; appropriate disease coding; global data sharing and federated patient registries that facilitate global clinical trials and research projects; and targeted social policies and legislation to encourage investment in and access to therapies for small population groups. Moreover, there are additional challenges to be overcome that will require large-scale systemic change and will benefit all individuals, not just those with rare diseases. This will require a cohesive and collaborative population-based approach driven by ethical decision-making approaches.

## Leveraging Precision Medicine for Precision Public Health

**Figure 1:**
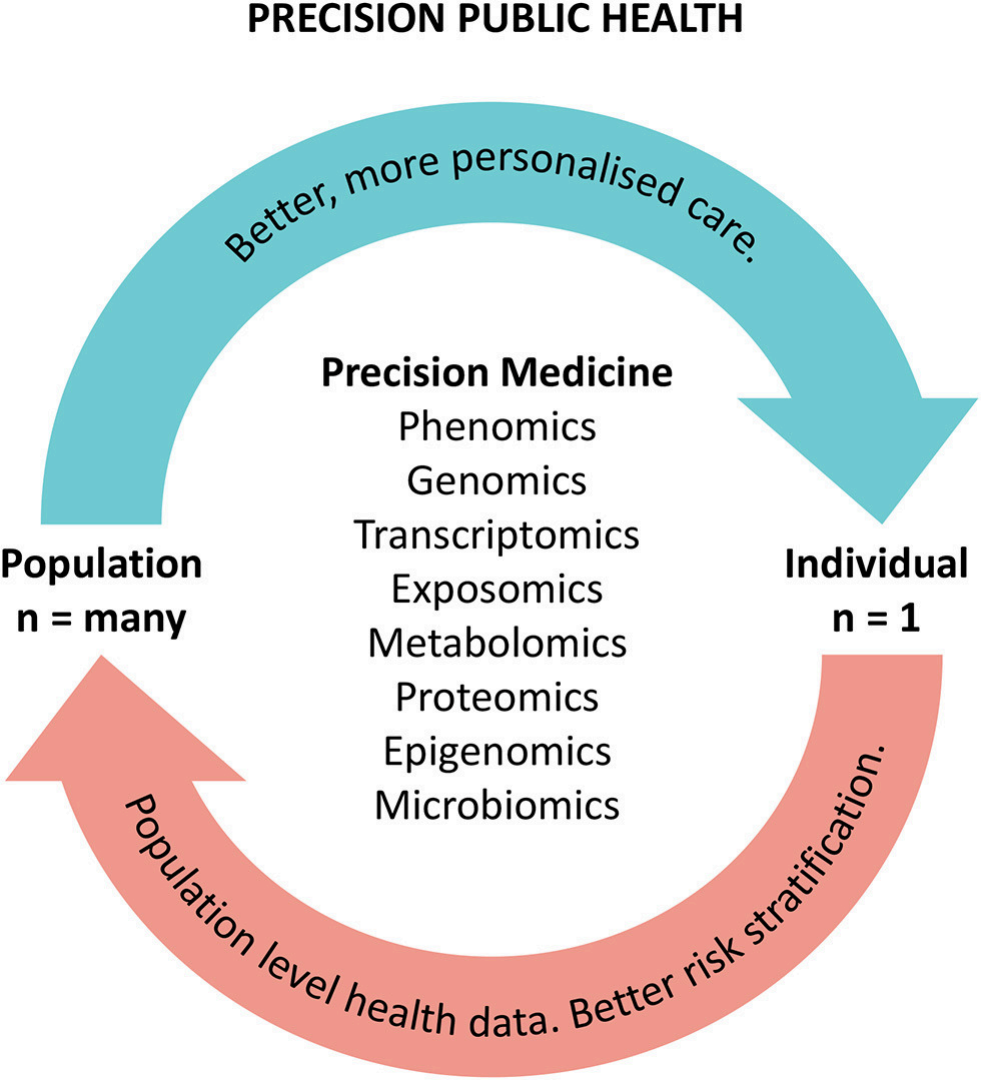
The precision public health cycle. The cycle illustrates the benefits of precision approaches to improving patient care and population health.

These authors believe the solution in reconciling the *n of 1* with the *n of many* approach for precision medicine and public health respectively lies within using precision medicine technologies to more accurately identify and define population cohorts, through increased understanding of the underlying causes and biological pathways of disease and health. That is, improved molecular understanding of disease and the underlying biological pathways create new knowledge that unlocks opportunities for discovery and re-aggregation of patient cohorts. The benefits of this approach include drug repurposing, new therapies, and stratification into new clinical pathways. Aggregation of population cohorts based on commonalities in biological pathways could therefore unlock efficiencies in diagnostic and therapeutic approaches and improve equity of access to precision medicines. A highly functioning PPH system will deliver benefits from technologies such as better collective understanding of phenomics, genomics, and other “–omics” (such as proteomics, metabolomics, and exposomics) to enable more precise care for individuals. Crucially, our understanding of individual pathologies and biological pathways will also unlock data and knowledge for our population, allowing a PPH approach (5, 56) (see Figure 1).

There are a number of key enablers that will be important in applying a PPH approach to integrating precision medicine for populations. One of these enablers is precise, accurate and timely data with digitally-enabled health information systems. Big data drawing on all of the collective learnings from individual precision medicine applications can be utilized to inform decisions around how precision therapies can be delivered on a population level. This is a cyclical feedback loop where big data captured through PPH can then lead to better and more precise individual therapies, resulting in better health for both individuals and populations.

Globally, initiatives such as the “All of Us Research Program” in the USA that is collecting data from one million volunteers, are moving toward integrated data collection to better inform public health system initiatives and precision medicine (57). Such data include information about genomes, societies and behaviors to add insight into the prevention and treatment of disease, whilst also hoping to uncover new ways to reduce health disparities (58). Similarly in the United Kingdom, the 100,000 Genomes Project was established to develop the infrastructure and workflows to enable clinical whole genome sequencing on a grand scale. This project focuses on patients and their families with rare diseases, and patients with cancer, and not only links with research and provides longitudinal data, but also expedites molecular testing as part of NHS clinical care (59). These large-scale national collaborations are leaving a legacy of capability, with workflows and technology being established to enable precision medicine to occur on a scale that will inform better healthcare delivery for all. Importantly, embedding this kind of technology into public health information systems will ensure that the values of equity and access are upheld.

Decision-makers and government may tackle some of the more unique ethical issues surrounding precision medicine approaches through the use of expert forums or working groups. Such groups could develop guidelines and processes around the use of precision medicine that reflect societal values and the specific needs of the community. To truly unlock the potential for precision medicine approaches to transform the health of the population, participation and engagement of the community and the public is imperative (60, 61). The community voice must drive the PPH movement through understanding the community's priorities for action, as well as understanding and responding to barriers of engagement.

Even with favorable economic evaluations, supportive social values and an advantageous political agenda, the integration of precision medicine into the healthcare system will require foresight to ensure systems and processes are in place for sustainable and equitable delivery. Countries without the computational or data infrastructure to support the collection and analysis of large datasets will require investment in these areas. The additional workforce expertise and workflows created with new clinical pathways will require collaboration and global data sharing, mapping and monitoring to ensure longevity. Such workforce considerations include enhancing the understanding of precision medicine and genomic literacy of the healthcare and public health workforce (62, 63), and investment in laboratory technology and bioinformatics expertise (64). In addition, leveraging the expertise of data scientists and upskilling public health practitioners to utilize big data will be necessary to ensure that precision approaches are translated successfully into public health programs.

The examples of uncommon cancers and rare diseases exemplify some longstanding issues that will be faced increasingly as precision medicine is applied more broadly to population health through other fields, such as pharmacogenomics, precision psychiatry, or microbiomics. As with rare diseases, large, national and often international collaborations will be required to enable robust efficacy and economic evaluations to guide healthcare decision-making and investment in the precision era. In this era, multi-sectorial research perspectives, including health economics and health services research and approaches, must collaborate and evolve alongside therapeutics to ensure effective translation of research efforts into equitable public health initiatives (65).

Population-level data and knowledge will enable population-based public health programs that accurately identify and stratify population cohorts and will in turn enable greater benefits from precision medicine for all individuals. PPH acknowledges the possible inefficiencies in targeting whole populations in the same way for particular health outcomes, instead embracing the possibilities that advances in technology, genomic and other “-omics” knowledge and data capability may lead to more efficient allocation of healthcare resources to better target populations in need (66). A recent example where a new approach has improved outcomes for a patient population was demonstrated in an RCT of the use of pharmacogenomics testing to guide prescription of therapeutics in patients with major depressive disorder. In this patient population, response and remission outcomes were improved when pharmacogenomics testing assisted prescription of anti-depressants compared with standard care (67).

The aggregation of smaller datasets to demonstrate the efficacy of a precision medicine approach (such as molecular tumor sequencing for all oncology patients) rather than focusing on individual subtypes and novel agents, may mitigate the hurdle of small patient numbers preventing the individual demonstration of economic efficiency. New transformative trial designs have been developed to address the challenges of assessing the efficacy of precision therapies, which target the molecular profile of a disease. Basket trials are an innovative clinical trial design for evaluating targeted therapies across different tumor types through grouping patients based on molecular markers independently of their tumor histology (68). An alternative approach is the umbrella trial where patients with the same tumor type are assigned to different treatment arms based on molecular markers (69). Furthermore, adaptive trial designs provide more efficient mechanisms for assessing precision medicine therapies, through changing key components of the trial design during implementation, while retaining its scientific validity. This allows multiple research questions to potentially be answered at once, meaning that multiple precision therapies can be assessed at once (70). The design of an adaptive trial evolves dynamically based on the efficacy data collected during the trial; randomization ratios can be changed, treatment arms can be dropped and/or added and a biomarker selection strategy can be changed even when treatment assignment remains the same (71). Adaptive trials have the potential for decreased time to completion, reduced resource requirements and number of patients exposed to inferior treatments, and overall likelihood of trial success (72).

**Figure 2:**
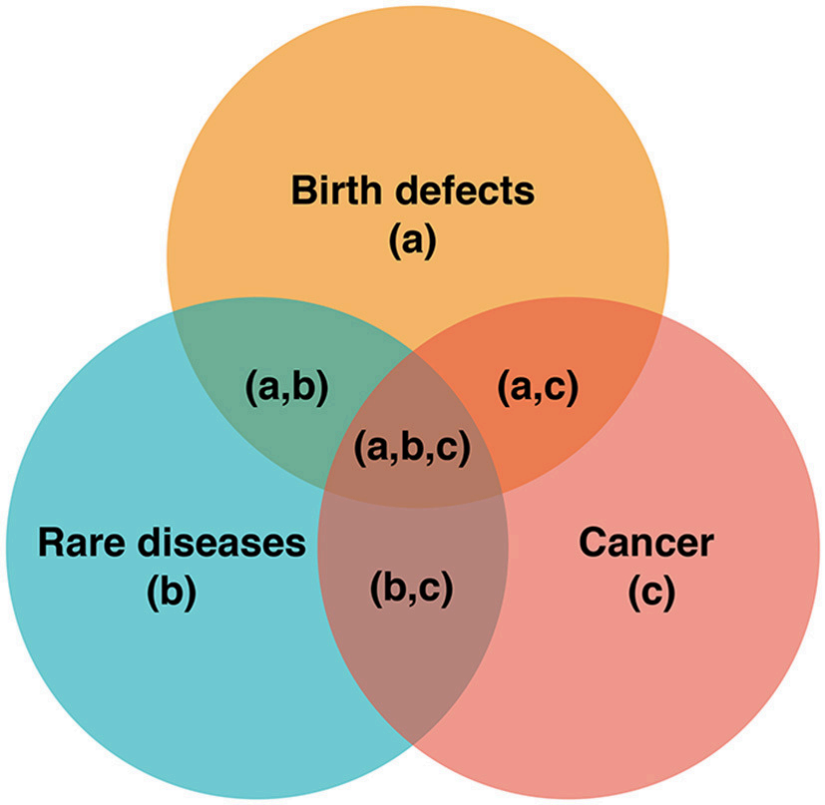
Pathways, intersections and precision medicine. The intersections of (a), (b), and (c) represent opportunities for better risk stratification, surveillance, and therapeutics. For example, understanding the biological pathways resulting in the intersecting pathologies at (a,c), (a,b,c), and (b,c), can facilitate improved cancer surveillance (e.g., cancer screening).

A broader example of the PPH approach, which bridges precision medicine and public health, is the discovery of overlap in understanding of birth defects, cancer, and rare diseases, such that previously distinct disciplines now intersect (see Case Study 1, Figure 2). While individuals with such conditions were previously categorized into three different disease groups, new knowledge from precision medicine allows better stratification of risk (e.g., the risk of an individual with a rare disease going on to develop cancer), new surveillance pathways, and new therapeutic options for identified patients. Better understanding of the pathogenesis of diseases within these three areas translates to better, more precise healthcare for patients with birth defects, cancer, and rare diseases. This new knowledge can be utilized to aggregate populations, better targeting health initiatives and fulfilling the PPH paradigm. Not only are there intersections for birth defects, rare diseases, and cancer, there is also an opportunity to identify more targeted prevention and surveillance based on more accurate knowledge of disease risk profiles (see Case Study 2).

Box 1 **Case study 1. Biological intersections: insights from the *n of 1* unlocking knowledge for the *n of many*.**

Collaborative international knowledge sharing helped discover the pathogenic gain-of-function variant in the mammalian target of rapamycin (*MTOR*) gene in multiple affected children presenting with birth defects from the same Aboriginal Australian family. This rare disease is now referred to as MINDS syndrome (macrocephaly intellectual disability neurodevelopmental disorder-small thorax syndrome), reflecting its multisystem nature and its associated components. MTOR is a critical component of the RAS-MAPK pathway and is also at the intersection of other biological pathways implicated in birth defects, rare diseases and cancer. Based on the phenotype of the affected Australian children, and the new and definitive knowledge of the underlying biological pathway, the children were anticipated to be at an increased risk of cancer and therefore placed on a new clinical pathway (tumor surveillance for intra-abdominal tumors of the solid viscera). Also, drug repurposing (MTOR inhibitors) became a possible treatment option for (pre)-cancerous lesions as well as neurocognitive endpoints. Subsequently, other families with the same syndrome were described, including one with early onset bowel polyps (73) that were managed by colonoscopic removal and surveillance; this has now become a risk management option for other families with this condition. The affected children have a characteristic facial phenotype and 3-dimensional facial analysis has been used to monitor MTOR inhibitor therapy (74), perhaps suggesting a new objective monitoring tool (75) for this and other families with MTOR-associated, and biologically related, conditions.

Box 2 **Case Study 2. Translating precision knowledge at the population level to better identify and stratify public health programs.**

Cancer is amongst one of the leading causes of death globally, with a growing burden predicted to produce >18 million new cases of cancer and almost 10 million cancer deaths worldwide in 2018 (76). A significant proportion of mortality and morbidity from cancer is due to late diagnosis, when surgical and pharmacologic therapies are less effective (77). Therefore, timely identification of cancer can be crucial and highly beneficial, detecting cancers before they spread, and allowing precision medicines to be delivered as early as possible.

Due to this knowledge, cancer screening programs have as their aim the detection of cancer before it has developed or before symptoms arise. Current population-based cancer screening programs have target demographics based on age and/or sex [e.g., the national bowel, breast and cervical cancer screening programs in Australia (78)]; with the success of these programs recently described (79). Yet how might such screening programs be improved to better mitigate the growing cancer burden, and how might screening programs for other as yet unscreened cancers be developed? Is it possible to stratify populations beyond traditional groupings such as age and sex; are there emerging tools to detect the currently screened cancers earlier than the methods presently utilized; can emerging tools detect types of cancers that have previously not been effectively detected at an early stage; and once a person is identified with a cancer, how can the best therapy for them be known and then accessed? In other words, how can precision medicines be most effectively delivered to all those in the population who require them?

To address the great need to detect, diagnose, prognosticate and monitor cancers, accurate biomarkers have been extensively studied, with a focus on non- or low-invasiveness. Cell-free tumor DNA, created from apoptotic or necrotic tumor cells, or circulating tumor cells, has become a promising target for liquid biopsies (e.g., blood, urine, semen). Promising results have been achieved with the application of liquid biopsies for detection, therapy response and prognosis in clinical oncology [reviewed in (80)], for a range of cancers including pancreatic (81, 82), Ewing sarcoma and osteosarcoma (83), urothelial (84), lymphoma (85), and other hematological cancers (86). Some assays are able to detect multiple cancers, and prospective, multi-site observational clinical trials are ongoing (e.g., The Circulating Cell-free Genome Atlas Study; NCT0288978). CancerSEEK has a specificity >99% for eight different cancer types in asymptomatic individuals and a sensitivity range of 69–98% for five cancers with no existing screening tests for the general population (87). However, health economic evaluation of liquid biopsies is still in early stages (88).

Could liquid biopsies be integrated into current population-based cancer screening programs to provide even earlier detection of these cancers? Moreover, could there become a single, population-based cancer screening program able to detect a range of cancers with the same test, and indeed using a test that is easily and widely usable (e.g., even in remote regions with no specialized equipment)? Additionally, could it be possible to further stratify a target population to allow more tailored screening frequencies, so that those with a greater risk (e.g., those who have tested positive by oncogene panel testing; those with known high environmental exposure) are more frequently screened than those at less risk? A precision approach such as this is already being implemented for cervical cancer screening in Australia, with results from a human papillomavirus (HPV) test informing risk stratification of participants into different screening pathways.

Universal access to early diagnosis and subsequent accessibility to appropriate treatment for cancer is critical. A PPH approach to this problem would incorporate evidence-based technological advances, big data collection and analysis, and public health paradigms to ensure equity and effectiveness. The aim would be to facilitate everyone within a population (the *n of many*) receiving appropriate screening methodologies (both test type and test frequency) to detect the largest possible range of cancers at the earliest phase, and then offer all people with cancer the precision medicine most suited to them (the *n of 1*).

## Conclusion

Genomics and other-omics knowledge and technologies are transforming the way healthcare can be delivered through greater understanding of disease detection and therapeutics. Responsible decision-making in the climate of escalating healthcare costs is required to ensure that precision medicine can be properly tested on a scale to determine if this approach will lead to better patient outcomes. Additionally, traditional decision-making paradigms must be agile to the precision medicine approach to ensure knowledge and discovery can be translated effectively and efficiently for better patient care. Subsequently, decision-makers must determine if the goals of PPH can be met by equitably harnessing precision medicine approaches such that the right healthcare is delivered to the right population, at the right time, and in the right place.

## Author Contributions

GAB conceived the paper concept. All authors drafted the manuscript, provided critical input and approved the final manuscript version for submission.

### Conflict of Interest Statement

The authors declare that the research was conducted in the absence of any commercial or financial relationships that could be construed as a potential conflict of interest.
